# Statistical ensemble of gene regulatory networks of macrophage differentiation

**DOI:** 10.1186/s12859-016-1363-4

**Published:** 2016-12-22

**Authors:** Filippo Castiglione, Paolo Tieri, Alessandro Palma, Abdul Salam Jarrah

**Affiliations:** 1 Institute for Applied Computing, National Research Council of Italy, Via dei Taurini 19, Rome, 00185 Italy; 2 Department of Biology, University of Tor Vergata, Via della ricerca scientifica 1, Rome, 00133 Italy; 3 Department of Mathematics and Statistics, American University of Sharjah, P.O.Box 26666, Sharjah, UAE

**Keywords:** Macrophage differentiation, Gene regulatory network, Agent-based modelling, Multiscale modelling

## Abstract

**Background:**

Macrophages cover a major role in the immune system, being the most plastic cell yielding several key immune functions.

**Methods:**

Here we derived a minimalistic gene regulatory network model for the differentiation of macrophages into the two phenotypes M1 (pro-) and M2 (anti-inflammatory).

**Results:**

To test the model, we simulated a large number of such networks as in a statistical ensemble. In other words, to enable the inter-cellular crosstalk required to obtain an immune activation in which the macrophage plays its role, the simulated networks are not taken in isolation but combined with other cellular agents, thus setting up a discrete minimalistic model of the immune system at the microscopic/intracellular (i.e., genetic regulation) and mesoscopic/intercellular scale.

**Conclusions:**

We show that within the mesoscopic level description of cellular interaction and cooperation, the gene regulatory logic is coherent and contributes to the overall dynamics of the ensembles that shows, statistically, the expected behaviour.

## Background

Recently, multiscale modelling - i.e., the integration of mathematical and computational models of processes at different spatial, temporal and organisational levels - earned its way as a growingly relevant resource to address immunological questions [1], after becoming a valuable support to examine diverse physiopathological processes and explore biological complexity [2].

In general, multiscale modeling refers to different models implemented at different scales of resolution used concurrently for the description and simulation of a system. When implemented in the biological field, each model usually describes mechanisms specific of one spatial and/or temporal scale, and it is integrated and intertwined with the other models, describing different scales, by input/output exchange and feedback.

Classifying biological processes into discrete levels can be a helpful yet approximate representation [3]. Such levels are usually categorised into microscopic (mainly molecular and intracellular events), mesoscopic (cell-to-cell, host-pathogen events) and macroscopic (tissue/organ/organism) scales [4, 5].

Multiscale immunological models, which fully fall within the extent of systems biology and computational system medicine [6], and of the Virtual Physiological Human initiative [7], can be general purpose models or also directly focus on a range of pathologies by tailoring the models on patient-specific immunological profiles, with the scope to evaluate the efficacy of treatments, and to enhance therapeutic regimens.

Among immune-specialised cells, the macrophage covers a paramount role: it is the most plastic cells of the haematopoietic system, present in all body tissues with large functional heterogeneity, central for development, homeostasis, tissue repair and immunity [8]. The work presented here is in particular aimed at increasing our understanding of pro- and anti-inflammatory processes in which macrophage responses are involved (e.g., cancer, obesity, arthritis, infectious diseases such as leishmaniasis, phagocytosis processes, among many others) [8], and to suggest improved treatments of such widespread immunological disorders.

On the same track of our previous work aimed at integrating Th1/Th2/Th17/Treg differentiation in an agent-based immune system model [9], our attempt targets at integrating the microscopic scale (sensing and processing of immune mediators, immune signalling and transduction pathways), here modelled by means of a gene regulatory network driving human macrophage polarisation into M1/M2 phenotypes (as better described in the following paragraph), with the meso- and macroscopic scale, modelled as an individual-based simulation of the immune system [10].

This article is organised as follows. In the next section we will give the biological background of the macrophage maturation and the way we take into account the genetic circuitry affecting the differentiation state. Then we will describe how we combined this molecular-level description with the upper-level (i.e., cellular) description of the main immune rules enacting the immune response. The resulting “statistical ensemble” is then checked against patterns observed in real conditions hence demonstrating the soundness, though essential, rationale of the differentiation network.

### Macrophages and their differentiation into the M1 and M2 subtypes

Myelomonocytic cells, derived from bone marrow precursors, are important components of the immune system and they can differentiate into macrophages [11–13]. Macrophages are remarkably versatile in their ability to recognise and respond to a wide range of molecules, expressing different surface and intracellular receptors, signal transduction components, chemokines and interleukins, and a variety of tryptophan metabolism pathways. They have potent endocytic, phagocytic, and secretory functions, able to engage upon contact with different cell types, such as macrophages themselves, microorganisms and chemical mediators [14]. Plasticity and diversity have long been known to be features of the monocyte-macrophage differentiation pathway. Phagocyte-mediated innate immunity also has a built-in adaptive component, and the ability to mount a polarised response is a reflection of this [15–17].

Mirroring T helper type 1 / type 2 (Th1-Th2) polarisation, two distinct states of polarised activation for macrophages have been proposed in the literature: the *classically* activated (M1) macrophage phenotype and the *alternatively* activated (M2) macrophage phenotype [18]. M1 macrophages have a typical pro-inflammatory function, inhibiting cell proliferation and expressing tissue damage activities, while M2 macrophages have anti-inflammatory functions, promoting cell proliferation and tissue repair. M1 macrophages often work together with Th1 lymphocytes, much as M2 macrophages together with Th2 lymphocytes, to produce typical immune responses.

The polarised form of a macrophage can be inferred by the stimulus that leads the macrophage to its functioning. A typical inflammatory stimulus derives from interferon gamma (INF- *γ* herein also indicated IFNg) produced by Th1 cells. IFNg is the main cytokine associated with the M1 polarisation, together with lipopolysaccharide (LPS), which is a component of Gram-negative bacteria, and the granulocyte macrophage colony-stimulating factor (GM-CSF). They can lead to a strong pro-inflammatory pattern of gene expression, determining the production of IFN- *β*, interleukins IL-12, IL-1, IL-1 *β*, tumour necrosis factor TNF and nitric oxide (NO).

The main stimuli that lead to a M2 polarised form of the macrophages are those from IL-4, IL-13, IL-10 and glucocorticoid hormones. In contrast to M1, M2 macrophages produce low IL-12 (indicated IL-12lo) and high IL-10 (IL-10hi), with an efficient phagocytic activity and anti-inflammatory functions.

### Gene regulatory networks

Gene regulatory networks modelling has been identified as a valid mean to understand the way cells integrate extracellular stimuli to activate cellular programs such as the differentiation whereby detailed kinetic information is not available [19].

The typical study includes the following steps:

1. The most important signal transducers, transcription factors and target genes proven to be involved in the activation of a certain cellular program (such as the differentiation of the macrophage, in our specific case) are identified and their mutual relationships in terms of inhibition/activation is derived from existing literature or experimental data.
2. The resulting information is arranged in a graph, or network, (*N,E*) in which the nodes *N* are the molecules and the edges *E*=*E* ^−^∪*E* ^+^ are the relationships (i.e., an activation or enhancement or upregulation is indicated with an edge in the set *E* ^+^, whereas an inhibition is indicated by an edge in *E* ^−^).
3. A set of values representing the activation level or the concentration of the gene/molecules in *N* is given. For instance the simplest case is the Boolean one in which 0 indicates low concentration/no activation, and 1 indicates high concentration or activation. The multi-valued case is when *n*_*k*_ can take up more than two levels of activation/concentration.
4. For each node *n*_*k*_∈*N* it is defined the function *F*(·) (e.g., a Boolean function *F*:{0,1}^|*N*|^↦{0,1}^*N*^) of the incident edges specifying how the state of the node *n* at a certain time *t* is determined by the nodes *n*_*j*_ for which there exists an edge *e*_*jk*_∈*E*. This identifies the system as a dynamical system in which each node at time *t* is updated on the basis of the values of the nodes at the previous discrete time step.

Once established the network’s Boolean function *F*(·), the network is studied in terms of its dynamical properties in particular in the nature and number of steady states that the dynamics can attain since they are interpreted as stable patterns of gene expression that characterising the differentiation fate of the cell.

As for the function *F*(·), in the present work, we use the simple map

$$\begin{aligned} \forall i&=1,\dots,\vert N\vert \qquad x^{t+1}_{i} = F({x^{t}_{1}},\dots,x^{t}_{\vert N\vert})\\& \quad= \left({ \bigvee_{j\in E^{+}_{i}} {x^{t}_{j}}} \right) \wedge \lnot \left({ \bigvee_{j\in E^{-}_{i}} {x^{t}_{j}}} \right) \end{aligned} $$

where $E^{+}_{i} = \left \{ e_{u,v} | (v=i) \wedge (e_{u,v}\in E^{+}) \right \}$ is the set of activation arcs incident on node *i* and $E^{-}_{i} = \left \{ e_{u,v} | (v=i) \wedge (e_{u,v}\in E^{-}) \right \}$ is the set of inhibition arcs incident on node *i*. The function *F* states that a gene will become active if anyone of the activation gene is active and none of the inhibition genes is active. It all other cases the gene will remain or become inactive.

## A minimalistic view of the regulatory logic of macrophage differentiation

By following the steps described above, we inferred the regulatory network that controls the polarisation of macrophages from experimental data derived from literature. These data refer to the main molecules involved in the control of macrophages polarisation into form M1 and M2.

The M1 and M2 states represent cell activation states polarised by cytokines, initially determined using IFN- *γ*, LPS and IL-4 and IL-10 that are typically secreted by Th1 or Th2 cells, bacteria and B lymphocytes. The M1 status is polarised by Th1 cytokines and pro-inflammatory chemical mediators including several pathogen-associated molecular components. In contrast, M2 status is polarised by IL-10 and IL-4 [20]. An initial stimulus leads to the activation of specific transcription factors that mediate the changes in the target genes expression.

### Pro-inflammatory M1 subtype

Both the type 1 IFN (IFN- *α* and IFN- *β*) receptors and the type 2 IFN (IFN- *γ*) receptors have multi-chain structures, which are composed of at least two distinct subunits: IFNGR1 and IFNGR2 for the type 2 IFN receptor. Each of these receptor subunits interacts with a member of the Janus activated kinase (JAK) family [21]. In the case of the IFN- *γ* receptor, the IFNGR1 subunit is associated with JAK1, whereas IFNGR2 is associated with JAK2. The first step in the IFN- *γ* mediated signalling is the activation of these receptor-associated JAKs after a ligand-dependent modification and dimerisation of the receptor subunits, followed by autophosphorylation and activation of the specific JAKs, which then activate the classical JAK/STAT (signal transducer and activator of transcription) signalling pathways [22]. Following the stimulation of the IFN- *γ* receptor and the phosphorylation of JAK, what occurs is a dimerisation of STAT1, which binds as a homodimer to *cis regulatory elements* known as “gamma-activated sequences” (GAS) in the promoters of the genes encoding NOS2, the Major Histocompatibility Complex (MHC) class II transactivator (CIITA) and SOCS3 among others [21, 23, 24].

The immunocompetence of macrophages regulated by IFN- *γ* and JAK/STAT pathway corresponds to an increase in IFN- *γ* production. These molecular components are regulators of M1 polarisation, and they lead to the synthesis of cytokines, nitric oxide, reactive oxygen intermediates (ROI) and enzymes that participate in tissue remodelling [25]. LPS is a component of Gram-negative bacterial cell wall and induces the expression of a variety of genes that drives the innate immune response to bacterial infections. LPS signals through toll-like receptor TLR4 especially expressed on macrophages and neutrophils. TLRs mediate the response to a variety of infectious agents and facilitate induction of many pro-inflammatory genes [26, 27]. Signalling through TLR4 induces rapid activation of two distinct intracellular signalling pathways that mediate the activation of specific transcription factors, such as NF-kB via the MyD88-dependent pathway; these pathways converge to activate the transcription of NOS2, the inducible NO synthase [28–30]. M2 macrophages exhibit a functionally distinct phenotype to that of M1s, thanks to IL-4 and IL-13, which are able to induce the mannose receptor (MR) expression, and another cytokine, IL-10, that play a pivotal role in the M2 polarisation. It is well established that IL-4 is associated with Th2 responses, which have well-defined effects on macrophages, other cells and immune functions. IL-4 is a cytokine produced mostly in allergic, cellular and humoral responses to parasitic and extracellular pathogens. It upregulates the expression of the mannose receptor and MHC class II molecules by macrophages, which stimulates endocytosis and antigen presentation, and induces the expression of distinct chemokines [31]. IL-4 acts through signal transducer and activator of transcription 6 (STAT6).

### Anti-inflammatory M2 subtype

IL-10 produced by B cells drives the macrophage to a M2 phenotype. It acts on a distinct plasma-membrane receptor to those for IL-4 and IL-13 [32], and its effects on macrophage gene expression is different, involving several inhibition and effector functions, together with the activation and expression of distinct genes. IL-10 triggers a M2 macrophage polarisation with a characteristic cytokine pattern of expression of IL-10hi, IL-12lo, IL-23lo and TGF *β*+, which is associated with anti-inflammatory responses, immune regulation, tissue repair and tumour promotion. IL-10 is able to suppress the immune activation by down-regulating the expression of MHC II and pro-inflammatory cytokines, thus affecting M1-derived cytokines [33]. IL-10 is a potent signal transducer and activator of transcription 3 (STAT3)-dependent inhibitor of pro-inflammatory cytokine production and nitric oxide release. The upregulation of expression of IL-4R *α* (indicated IL-4Ra) by IL-10 is associated to increased levels of arginase-1 (Arg1) derived from the IL-4 pathway.

Peroxisome proliferator-activated receptor *γ* (PPAR *γ* herein PPARg) is a master regulator of lipid metabolism in macrophages and inhibits pro-inflammatory gene expression through several mechanisms, including the repression of NF-kB [34, 35]. PPARg is constitutively expressed by adipose tissue macrophages, but its expression can also be induced by IL-4 via STAT6, which indicates that M2 polarisation might also involve PPAR *γ*. Indeed, a recent study has shown an important role for STAT6 as a cofactor in PPAR *γ*-mediated gene regulation in vitro; therefore, crosstalk between PPAR *γ* and the IL-4/STAT6 axis might coordinately control the M2 phenotype [23].

IL-4-induced macrophage polarisation also involves Krüppel-like factor 4 (KLF4), which promotes M2 polarisation cooperating with STAT6 and blocking the M1 polarisation through the inhibition of NF-kB [36, 37].

Suppressor of cytokine signalling proteins (SOCS) play an important role in regulating M1 and M2 macrophage polarisation. SOCS1, induced by STAT6, promotes the M1 polarisation by inhibiting the JAK2/STAT1 signalling, while SOSC3, induced by STAT1, supports a pro-inflammatory phenotype inhibiting STAT3 [38].

**Fig. 1:**
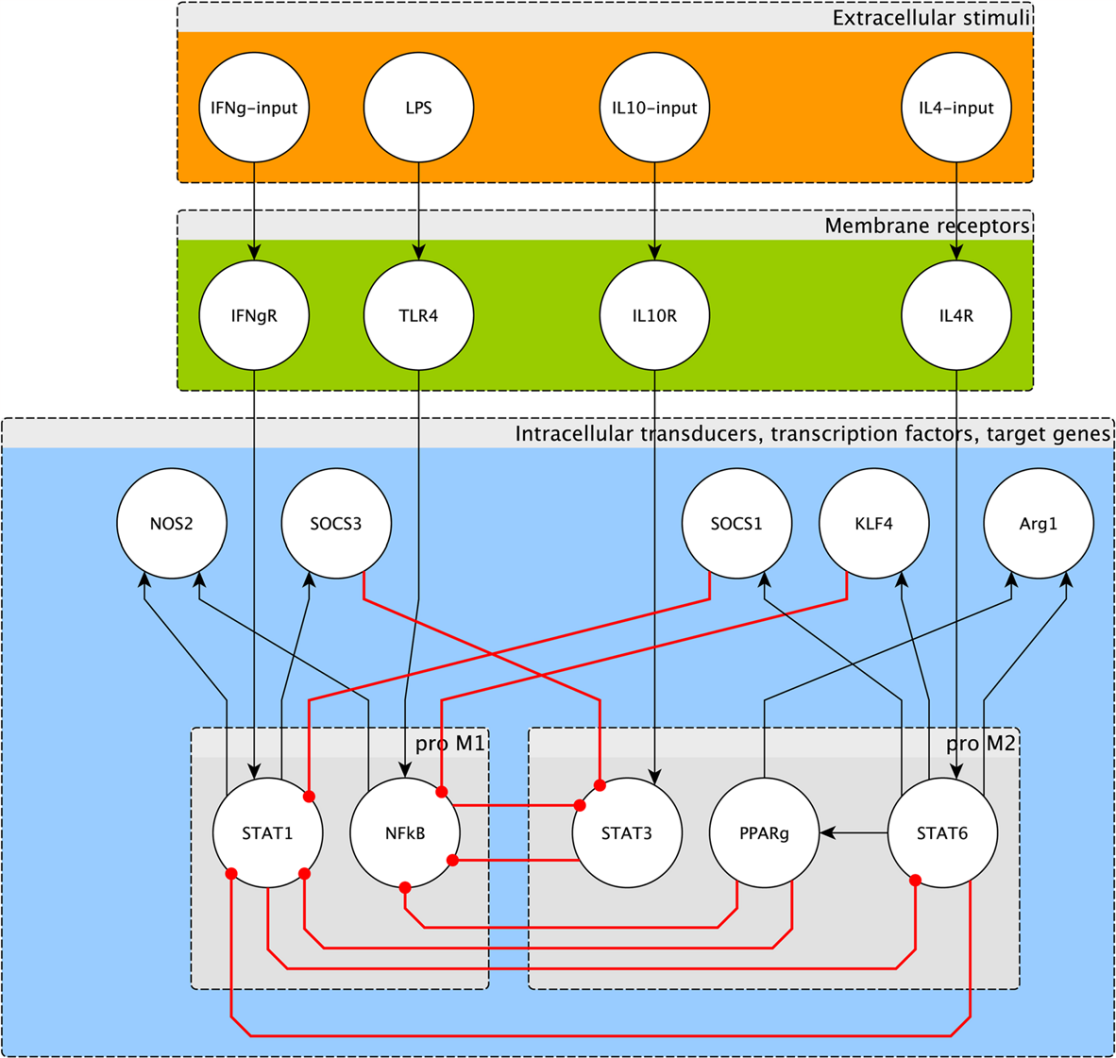
The gene regulatory network. A minimalistic gene regulatory networks of macrophage differentiation into the M1 and M2 subclasses. Signalling stimuli are “sensed” by membrane receptors that activate genes through the regulatory circuitry. Also see Table 1

Given the knowledge reported above we established the gene regulatory network (GRN) described in Fig. 1 and Table 1.

**Table 1:** Nodes of the network

Input	Receptor	Internal nodes	Phenotype	References
LPS	TLR4	NF-kB, NOS2	M1	[20–23, 25–30, 38]
IFNg	IFNgR	STAT1, SOCS3, NOS2	M1	[20–23, 25–30, 38]
IL-4	IL-4Ra	STAT6, PPARg,	M2	[23, 24, 31–38]
		SOCS1, KLF4, Arg1		
IL-10	IL-10R	STAT3	M2	[23, 24, 31–38]

Internal nodes means transducers/transcription factors/target genes

## Methods

### Statistical ensemble of gene regulatory networks

In statistical mechanics, a statistical ensemble refers to a large number (ideally infinite) of virtual copies of a system, each of which represents a possible and realistic state of the actual system. An ensemble therefore represents a probability distribution for the state of the system [39].

We generated copies of the above-constructed gene regulatory network of M1/M2 differentiation (i.e., the “system”) to build a statistical ensemble in order to calculate the probability distribution of the “state” of the differentiating macrophage, and to describe how this state changes with respect to biological stimuli such as bacterial infections or others.

Since a macrophage taken in isolation from the other immune cells and antigenic stimuli would not undergo any differentiation, we also incorporated in the model other cellular and molecular components so to have the main signals needed to represent both possible M1 and M2 differentiation pathways. This inclusion fully implements the mesoscopic (cell-cell interactions) of the model. The intra-cellular microscopic gene regulation level and the inter-cellular mesoscopic level, together constitute a multiscale system and enacts a full fledged adaptive immune response.

It is worth to note that for simplicity the model considers antigen-specific clonotypes of lymphocytes, i.e., it does not implements a full clonal selection process with antigen recognition.

In more details, we included in the ensemble virtual copies of B-cells (indicated *B*), macrophages (*M*), T helper lymphocytes (*T*), a generic antigen (*Ag*) bearing LPS as membrane molecules and cytokines IFNg (*IFNg*), IL-10 (*IL*10) and IL-4 (*IL*4).

Macrophages are subdivided in the undifferentiated phenotype (*M*_0_), in the terminally differentiated type-1 (indicated *M*_1_) and type-2 (*M*_2_).

Helper T lymphocytes are further divided in type-1 (indicated *H*_1_), type-2 (*H*_2_) and regulatory cells (*H*_*r*_) each of which secretes a different differentiation stimuli upon contact with the macrophage. Moreover, since cytokines are secreted by cells in particular activation states, we refined the representation of macrophages and lymphocytes including this information. Hence we defined macrophages in the resting (*M* ^*r*^), active (*M* ^*a*^) and presenting state (*M* ^*p*^); helper lymphocytes (all three classes 1,2,*r*) in the resting ($H_{1,2,r}^{r}$) and active ($H_{1,2,r}^{a}$) state; B lymphocytes in the active (*B* ^*a*^) and presenting state (*B* ^*p*^). By “presenting” we mean that the antigen presenting cell B or macrophage has captured, internalised, processed and presented the antigen on the cell membrane together with the Major Histocompatibility Complex molecule. This process is not further detailed to keep the model simple. Table 2 contains a symbol legend.

**Table 2:** Cell states

Cell type/state	Symbol
Macrophage resting or inactive	*M* ^*r*^
Macrophage active	*M* ^*a*^
Macrophage presenting the antigen	*M* ^*p*^
B-cell active	*B* ^*a*^
B-cell presenting the antigen	*B* ^*p*^
T-helper resting or inactive	*H* ^*r*^
T-helper active	*H* ^*a*^

*M* indicates both type-1 and -2 macrophages. Likewise *H* indicates type-1, -2 and regulatory T hyper lymphocytes

The so constructed mesoscopic cell interaction level can be seen as a *bioreactor* in which each individual cell undergoes state transformations according to predefined immunologically-motivated rules [40]. Cells populate a 3D lattice where they freely diffuse and interact according to stochastic rules of the form

$$\alpha A + \beta B + \cdots \overset{p}{\longrightarrow} \gamma G + \delta D + \cdots $$

meaning that the rule applied to *α* instances of A, *β* instances of B etc. that are located in the same lattice site will produce *γ* instances of G, *δ* instances of D etc. with probability *p*.

The full list of rules is the following:

1 $$\begin{array}{*{20}l} M^{r} + IFNg & \overset{p_{1}}{\longrightarrow} M^{a}  \end{array} $$

2 $$\begin{array}{*{20}l} M^{a} + Ag & \overset{p_{2}}{\longrightarrow} M^{p}  \end{array} $$

3 $$\begin{array}{*{20}l} M^{p} & \overset{p_{3}}{\longrightarrow} M^{a}  \end{array} $$

4 $$\begin{array}{*{20}l} M^{a} & \overset{p_{4}}{\longrightarrow} M^{r}  \end{array} $$

5 $$\begin{array}{*{20}l} M^{p} + {H_{1}^{a}} & \overset{p_{5}}{\longrightarrow} M^{p} + {H_{1}^{a}} + IFNg  \end{array} $$

6 $$\begin{array}{*{20}l} M^{p} + {H_{2}^{a}} & \overset{p_{6}}{\longrightarrow} M^{p} + {H_{2}^{a}} + IL4  \end{array} $$

7 $$\begin{array}{*{20}l} {M_{2}^{p}} + {H_{r}^{a}} & \overset{p_{7}}{\longrightarrow} M^{p} + {H_{r}^{a}} + IL10  \end{array} $$

8 $$\begin{array}{*{20}l} B^{a} + Ag & \overset{p_{8}}{\longrightarrow} B^{p}  \end{array} $$

9 $$\begin{array}{*{20}l} B^{p} & \overset{p_{9}}{\longrightarrow} B^{a}  \end{array} $$

10 $$\begin{array}{*{20}l} B^{p} + {H_{1}^{a}} & \overset{p_{1}0}{\longrightarrow} 2 B^{p} + 2 {H_{1}^{a}} + IFNg + Ab  \end{array} $$

11 $$\begin{array}{*{20}l} B^{p} + {H_{2}^{a}} & \overset{p_{11}}{\longrightarrow} 2 B^{p} + 2 {H_{1}^{a}} + IL4 + Ab  \end{array} $$

12 $$\begin{array}{*{20}l} H_{1,2,r}^{r} + M^{p} & \overset{p_{12}}{\longrightarrow} H_{1,2,r}^{a} + M^{p}  \end{array} $$

13 $$\begin{array}{*{20}l} H_{1,2,r}^{a} & \overset{p_{13}}{\longrightarrow} H_{1,2,r}^{r}  \end{array} $$

14 $$\begin{array}{*{20}l} {H_{1}^{a}} + B^{p} & \overset{p_{14}}{\longrightarrow} {H_{1}^{a}}+ B^{p} + IFNg  \end{array} $$

15 $$\begin{array}{*{20}l} Ab + Ag & \overset{p_{15}}{\longrightarrow} \emptyset  \end{array} $$

The first “reaction”, or rule R1 of Eq. (1), stands for macrophage activation by IFNg.

R2 represents antigen phagocytation, digestion and presentation by macrophages.

R3 stands for macrophages stopping presenting the antigen on cell surface.

R4 stands for macrophages returning to the resting state.

R5 indicates macrophage presenting the antigen to helper T cells of type-1 and inducing release of IFNg.

R6 is the same as before but for type-2 the released cytokine is IL-4.

R7 likewise, upon contact with *M*_2_ cells, Treg lymphocytes release IL-10.

R8 is antigen phagocytation, digestion and presentation by B-cells.

R9 stands for B-cells stopping presenting the antigen on cell surface thus going back to the active state.

R10 is B-cells presenting the antigen to helper T cells of type-1 and inducing release of IFNg as well as antibodies (with the implicit assumption of B-cell differentiation to plasma B cells secreting antibodies).

R11 is the same as before but for type-2 the released cytokine is IL-4.

R12 antigen presenting macrophage activation of helper T-cells.

R13 stands for helper T-cells going back to resting.

R14 is releasing of IFNg by class-1 T-helper upon recognition of antigen peptides presented by B-cells.

R15 is the neutralisation of antigen by the antibodies.

These rules are executed in randomised order at each simulation step (to avoid biases). In principle *p* should be different for each rule, however, to keep the model simple we set ∀*i*=1,…,15,*p*_*i*_=1, that is, we allow all reactions to take place whenever all the necessary molecules are met on the same lattice point.

We can say that the ensemble consists of a Reactive Lattice Gas Automata (RLGA) on a three-dimensional cartesian lattice *L*×*L*×*L* with periodic boundary conditions. At the start, a large number of copies of cells populate the simulated volume. The relative proportion of lymphocytes and monocytes is set as in the standard human white blood cell counts [41]. Macrophages, that are the only agents whose internal dynamics is fully specified by the network show in Fig. 1, are initialised with the gene-state equal to zero meaning that the nodes of the network are in the inactive form (see below)). The rules R1–R15 (respectively Eq. (11)–(15)) are then applied in each lattice point. After that, all entities diffuse randomly on the lattice with equal speed. In this respect the lattice represents a homogenous media and a lack of differences in the diffusion speed of the various cells and molecules does not affect the “logic” of the model, which is what we are more interested in studying.

### The rule for macrophage differentiation

The last rules of the mesoscopic model implements the differentiation of the macrophages from the undifferentiated *M*_0_ phenotype to either *M*_1_ or *M*_2_. They realise the connection between the two levels of the multiscale description of the model.

As already mentioned, the networks in the ensemble are initialised as $\bar {x_{0}} = \bar {0}$, that is, all genes are not expressed in the initially populating phenotype *M*_0_. The differentiation is then performed according to the following rule

16 $$\begin{array}{*{20}l} M_{0}^{a,p} & \overset{f_{1}}{\longrightarrow} M_{1}^{a,p}  \end{array} $$

17 $$\begin{array}{*{20}l} M_{0}^{a,p} & \overset{f_{2}}{\longrightarrow} M_{2}^{a,p}  \end{array} $$

where the macrophage has to be either active (superscript *a*) or presenting (superscript *p*) and the *f*_1_ and *f*_2_ are two probability functions defined as follows

$${} f_{1} \!\equiv\! \left\{ \begin{array}{lr} 1, & S(\bar{x}_{t} \wedge I,k) \,=\, X_{1}\\ 0, & \text{otherwise}\\ \end{array} \right. \qquad f_{2} \equiv \left\{ \begin{array}{lr} 1, & S(\bar{x}_{t} \wedge I,k) = X_{2}\\ 0, & \text{otherwise}\\ \end{array} \right. $$

with

$$S(\bar{x}_{t} \wedge I,k) = \underbrace{F \circ F \circ \cdots \circ F}_{\mathrm{k}} (\bar{x}_{t} \wedge I). $$

Thus, if the network state attained after *k* synchronous application of the Boolean dynamics *F*(·) reaches an attractor *X*_1_ or *X*_2_, then the macrophage is considered committed, that is, terminally differentiated to the type-1 or type-2. Otherwise the state remains $\bar {x}_{t+k}$ and the macrophages is still undifferentiated (i.e., *M*_0_). Specifically, $S(\bar {x}_{t} \wedge I,k)$ is the state of the network after *k* application of the function *F*(·) starting from $\bar {x}_{t}$ and after the input nodes have been updated with *I*=(*IFNg,IL*10,*IL*4,*LPS*) where LPS here means Ag with LPS on its surface membrane. In other words $\bar {x}_{t} \wedge I$ is the vector $\bar {x}_{t}$ with the values corresponding to IFNg, IL10, LPS and IL4 updated with the values in *I* (see extracellular stimuli layer in Fig. 1). Now if

$$\forall k>0, k\in \mathbb{N}, \quad S(\bar{x}_{t} \wedge I,k) = \bar{x}_{t} \wedge I $$

this means that the dynamics has converged to a fixed point. We call *X*_1_ the fixed point associated to the phenotype *M*_1_ and *X*_2_ the fixed point associated to the phenotype *M*_2_. By construction *X*_1_≠*X*_2_ and therefore it follows that in rules R16 and R17 (respectively in Eqs. (16) and (17)) it must be that at any time *f*_1_+*f*_2_≤1 (i.e., none or only one of the two steady state is reached). In other words, each network of the ensemble representing a macrophage initially in the undifferentiated state has the chance to differentiate at each discrete step on the basis of its current state $\bar {x}_{t}$ and the input *I*, which in turn is given by the local (i.e., in the same lattice site) “concentration” of IFNg, IL10, IL4 and antigen with LPS in the current lattice location (the lattice location has not been indicated in the formalism above for simplicity).

## Results and discussion

### Ensemble dynamics

Here we describe what we obtained when we challenged the system with different stimuli driving the differential polarisation of macrophages. To show it, we have identified a couple of situations in which there is experimental evidence about polarisation in one or the opposite direction, and verified that the in silico model is in agreement with those. The two cases are described below.

### M1 polarisation

**Fig. 2:**
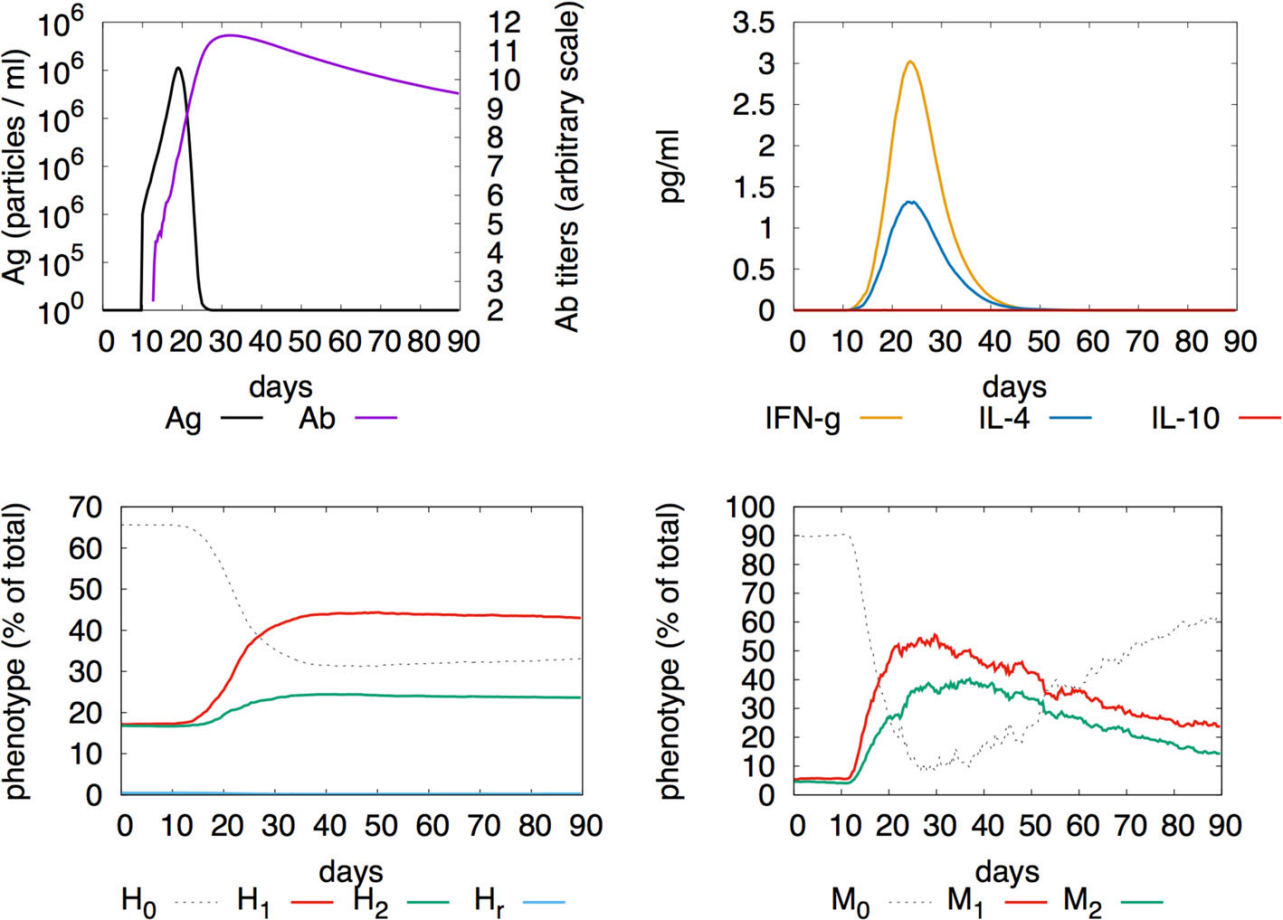
M1-polarised response. Challenging the system with a Gram-negative bacteria the system responds with a M1/H1 polarised immune response. The *top-left panel* shows bacterial growth and antibody production; *top-right panel* shows production of IFNg cytokines as well as to a smaller extend IL-4 and no IL-10; *bottom-left panel* shows polarisation of helper T-cells to *H*_1_; *bottom-right panel* shows polarisation of macrophages toward the *M*_1_ phenotype

We obtain a pro-inflammatory response if we challenge the system with an LPS-carrying (i.e., Gram-negative) bacterium. In this case a clear M1 polarisation is shown in Fig. 2 (more details can be found in the figure caption). This is in agreement with [12, 13] and represents the primary pro-inflammatory function of macrophages during bacterial infections.

### M2 polarisation

IL-4 drives the macrophage to a M2 phenotype, involving both innate and adaptive immune system cells. IL-4 is secreted during Th2 immune responses, after a disturbance at mucosal surfaces, especially in lung and intestines, or after fungi or helmint infections [42]. Indeed, in vitro experiments on macrophages treated with IL-4, showed a decreased production of pro-inflammatory cytokines and oxygen radicals [43]. Furthermore, basophils and mast cells are important early sources of IL-4, which is produced as one of the first innate signals to be released after injury, and in response to chitin, a typical product of fungi and parasites. As a result, an increasing of the wound healing macrophage population is observed, switching towards a M2-like phenotype, with the production of aginase and other typical anti-inflammatory molecules, and the secretion of extracellular matrix components [23].

**Fig. 3:**
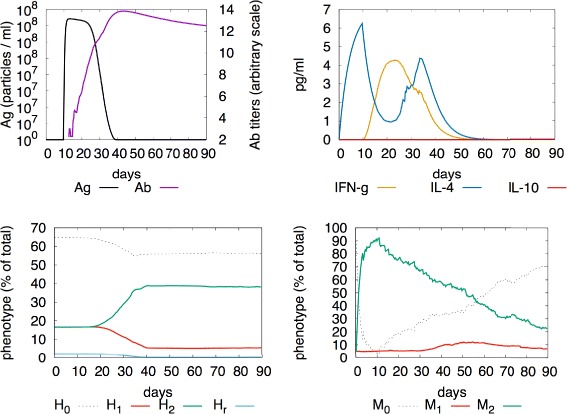
M2-polarised response. Here the system is challenged by a vaccine which does not include LPS as adjuvant (e.g., inactive gram positive bacteria). After a short while (after 3 days and for a whole week) we also inject the pro-M2 cytokines IL-4. In this case the M2-polarisation is shown in the *bottom right panel*, followed by a polarisation of T-helpers toward the *H*_2_ phenotype

In our model, when the systems is challenged with a simple vaccine which does not carry LPS membrane molecules but, after a short while (after 3 days and for a whole week) the pro-M2 cytokines IL-4 is injected (simulating basophils and mast cells contribution), an M2 polarisation is obtained (this is shown in Fig. 3).

IL-10 is a cytokine produced by all leukocytes, including macrophages, dendritic cells, natural killer cells, neutrophils, eosinophils, mast cells, B cells, and some T-cell populations. In macrophages, it acts as a potent anti-inflammatory molecule, playing a pivotal role in limiting immune-mediated pathology. In helminth infections, macrophages are an important source of IL-10. When produced by regulatory T cells, IL-10 determines the polarisation of regulatory macrophages, which act as antigen-presenting cells, produce IL-10 and can induce the expansion of Th2 cells. IL-10 is a potent downregulator of macrophage gene expression, modulating IL-4 and IL-13 and IFNg actions. Indeed, IL-10 has actions on macrophages that are clearly distinct from those of IL-4 and IL-13, which reflects the pattern of gene expression of IL-10-stimulated macrophages [11, 31, 43–45]

On the basis of what just reported, another possibility to trigger an M2 immune response in the computational model is to inject a bacterium (with LPS) followed by an injection of IL-10. This situation mimics the case in which IL-10 comes from sources other than macrophages, as for instance in allergies mediated through IgE signalling which triggers mast cell degranulation and release of IL-10 [46]. Indeed, many forms of cutaneous and mucosal hypersensitivity reactions are mediated in large part by mast cells. They play a central role in asthma, eczema, itch (from various causes), and allergic rhinitis and allergic conjunctivitis.

**Fig. 4:**
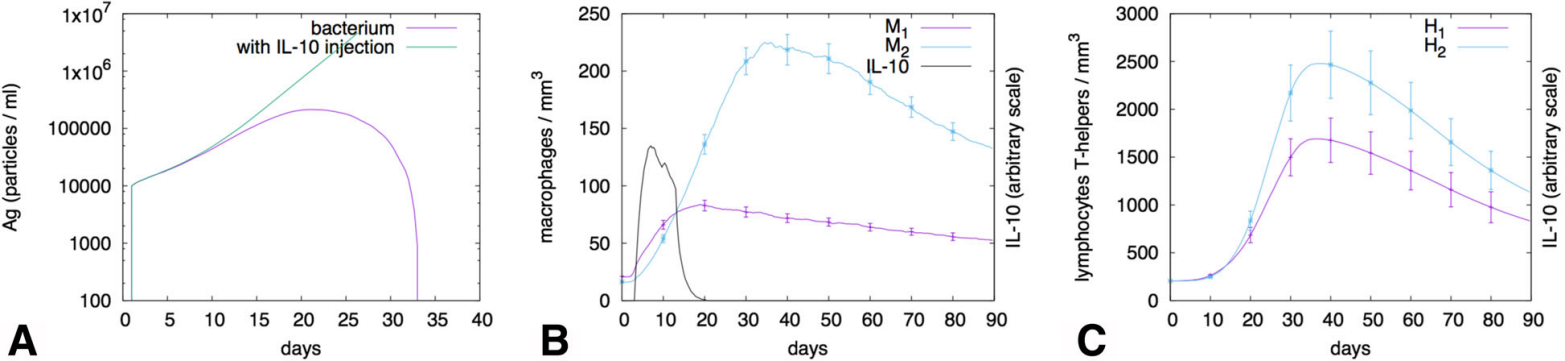
M2-polarised response. The effect of injecting IL-10 after a bacterial infection is clearly shown is panel **a** in two representative cases of possible outcome; in some cases (43% of total stochastic simulations performed) IL-10 dampens the immune response. Panel **b** shows the average population kinetics of the two macrophage sub-types as well as the injected IL-10 concentration (error-bars show standard deviation over a number of stochastic simulations). This plot demomstrates that IL-10 downregulates immune reponse by promoting M2 differentiation. Similarly, panel **c** shows T-helper cell sub-types

In this case in the model the initial *H*_1_/ *M*_1_ response shifts toward an *M*_2_ response which is ineffective compared to the previous as witnessed by the fact that the bacterium continues to grow rather being controlled (see Fig. 4 and caption for details).

## Conclusions

Macrophages play critical functions in the immune response development, homeostasis, tissue repair and immunity. Accordingly, a number of disorders in humans and mice have been related to deregulated macrophage differentiation process and cell function (e.g., leishmaniasis, neoplastic diseases, asthma and respiratory diseases, neuropathies, stroke, among others) [47]. The complexity and plasticity of the macrophage differentiation only recently have gained much appreciation.

Here we implemented a multiscale computational approach to simulate macrophage differentiation in which two different level of description, i.e., gene regulation and cell population dynamics are combined into a complex immune system model.

The model described here does not focus on a specific disease nor it encompasses pathogen-specific processes such as virus mutation or tumor escape for which a more detailed description of the immunological processes would be required as in the model described in [9, 10]. It shows however how such model integration allows bridging a gap between gene level information and cell level population by testing the model to reproduce behaviour qualitatively coherent with experimental data when exposed to different immunological challenges.

In our former work, we introduced Th1/Th2/Th17/Treg differentiation [9] in an integrated agent-based immune system model, further steps toward the enhancement of the integrated model will concern the merging and fine tuning of such different cell types combined dynamics.

The macrophage differentiation network presented here is minimalistic, although coherent with respect to the dynamics shown hence there is no reason to resort to stability analysis or other methods to understand its dynamical properties. We are currently constructing an enlarged version of the network with the aim of performing more detailed and extensive simulations including stability analysis and perturbation experiments such as knockouts and ectopic expressions, to see how they affect the network functionality. This work will proceed along the line of the method described in this article with the hope to better understand the complex role of the macrophages in the elicitation of the adaptive immune response.
